# Determinants of contraceptive discontinuation among sexually active women in Nigeria: a systematic review

**DOI:** 10.1186/s12889-026-28497-5

**Published:** 2026-07-13

**Authors:** Aanuoluwapo Afolabi, Olayinka Ilesanmi, Adamu Akyala

**Affiliations:** 1 Global Health and Infectious Diseases Institute, Nasarawa State University, Keffi, Nigeria; 2 MSI Nigeria Reproductive Choices, Abuja, Nigeria; 3 Africa Centres for Disease Control and Prevention, Addis Ababa, Ethiopia

**Keywords:** Contraceptive discontinuation, Family planning, Pregnancy, Reproductive health, Nigeria

## Abstract

**Introduction:**

Contraceptive use is critical for improving reproductive health and reducing unintended pregnancies, yet many women discontinue its use despite the need for it. In Nigeria, the total fertility rate remains high at 4.8 children per woman, while modern contraceptive prevalence among women of reproductive age is only 17–20%, highlighting persistent gaps in family planning. This systematic review aimed to synthesize evidence on the prevalence, patterns, and determinants of contraceptive discontinuation among Nigerian women.

**Methods:**

A systematic review was conducted focusing on studies from Nigeria involving sexually active women aged 15–49 years who had previously used contraceptives. Multiple databases were searched using keywords for family planning, discontinuation, and Nigeria. Eligible observational, qualitative, and mixed-methods studies were screened and selected using the PRISMA framework. Data extraction included prevalence, method-specific discontinuation, and reasons for cessation. After screening 12,768 records and removing duplicates, 48 studies were included in the review.

**Results:**

Discontinuation rates varied by method type. Condoms were discontinued primarily due to behavioral factors, such as inconsistent use, discomfort, or misconceptions, with 12-month discontinuation rates around 17–18%. Oral contraceptives were discontinued within the first 12 months by 21–59% of users, largely due to side effects, daily regimen demands, and personal fertility intentions. Injectables showed discontinuation rates of 19.8–53.3%, mostly within the first 3–6 months, driven by side effects and the desire for pregnancy. Long-acting reversible contraceptives, including implants and intrauterine devices, had lower discontinuation rates (7%–42.9% at 12 months), though early discontinuation was associated with menstrual irregularities, desire for pregnancy, or method-specific side effects. Key determinants of discontinuation included side effects, fertility desires, spousal opposition, cultural or religious beliefs, and poor-quality counselling.

**Conclusion:**

Contraceptive discontinuation in Nigeria undermines the effectiveness of family planning programmes and contributes to unintended high fertility rates. Policymakers should strengthen national counselling standards, while donors should invest in provider training, follow-up systems, and commodity supply and implementers, including healthcare providers and non-governmental organizations should prioritize client-centered counselling, management of side effects, and community engagement to support sustained contraceptive use. Coordinated action across these levels is essential to reduce unmet need and improve reproductive health outcomes.

## Introduction

Contraceptives have been widely recognized as essential tools for improving reproductive health, reducing unintended pregnancies, and lowering maternal and infant mortality rates [1, 2]. Over the past few decades, significant progress has been made in expanding access to modern contraception among women who desire to space or limit childbearing [3, 4]. Despite the availability and effectiveness of contraceptives, many sexually active women discontinue its use despite having a need to use it [5, 6]. This discontinuation is not a minor concern; it has far-reaching implications for women, families, and healthcare systems. When a woman stops using a contraceptive method without adopting alternative methods, she faces an increased risk of unintended pregnancies, which can lead to unsafe abortions, high-risk births, and socioeconomic challenges that impact not only her own well-being but also that of her family and community [7–9].

While much of the global conversation around contraception has focused on improving access and increasing uptake, less attention has been given to what happens after a woman begins using a contraceptive method [10–12]. Discontinuation disrupts the effectiveness of family (FP) programs [13] and raises critical questions: What proportion of women discontinue contraception within a given time frame? What are the patterns of discontinuation across different methods and demographics? After stopping a contraceptive method, do women switch to another method, or do they remain without protection? What are the long-term reproductive health consequences of discontinuation? These questions are especially pertinent in Africa, where contraceptive use remains lower than the global average, and fertility rates are among the highest in the world.

Research on contraceptive discontinuation is essential for informing policies and programs that aim to improve FP services [14]. While studies have examined contraceptive use broadly, there remains a gap in understanding the trajectories of women who discontinue their chosen methods [15, 16]. Without this knowledge, FP initiatives may fail to address the challenges that lead to discontinuation, ultimately undermining efforts to promote sustained contraceptive use. This systematic review sought to fill this gap by synthesizing existing research on contraceptive discontinuation among sexually active women in Africa. By analyzing patterns and trends, this review aimed to provide a clearer picture of the extent of discontinuation and its broader implications. The findings will offer valuable insights for policymakers, healthcare providers, and organizations working to strengthen reproductive health programs, ensuring that contraceptive use is not only initiated but also sustained over time for better health outcomes.

## Methods

### Study design

This study employed a systematic review design, which involves a structured, comprehensive, and transparent approach to identifying, selecting, and synthesizing relevant published literature. The goal of the review was to gather and critically analyze existing evidence on the research topic using clearly defined criteria and methodologies. The systematic review followed a stepwise process that included defining the research question and objectives, establishing inclusion and exclusion criteria for article selection, conducting a comprehensive literature search across multiple databases, screening and selecting eligible studies based on relevance and methodological rigor, extracting key data from included articles, assessing the quality of the studies using appropriate appraisal tools, and synthesizing the findings to draw conclusions based on the available evidence. This design ensured that the review was reproducible, minimized bias, and provided a reliable summary of the current knowledge landscape on the subject.

### Study area

This systematic review focused on studies conducted in Nigeria, the most populous country in Africa, which continues to experience a high total fertility rate of about 4.8 children per woman and a substantial unmet need for FP of approximately 21% according to the 2023–2024 Nigeria and Health Survey (DHS) [17]. Nigeria offers a critical context for exploring contraceptive discontinuation among women, as it is influenced by a combination of health system limitations, including inconsistent method availability, provider bias, and poor counselling quality, and deeply entrenched social and gender norms [18]. These factors vary widely across regions and cultures within the country, shaping women’s experiences with FP services. Limiting the review to Nigerian-based studies allowed for a nuanced understanding of the country-specific drivers of contraceptive discontinuation, and the ways in which health system practices and social expectations intersect to influence women’s decisions to stop using contraception.

### Population, exposure, and outcome elements

The population referred to sexually active women of reproductive age (15–49 years) in Nigeria.

Exposure was previous use and discontinuation of contraceptives, regardless of the method that was discontinued.

The outcome referred to the reported reasons for discontinuation, as documented in published literature.

### Search strategy

The search strategy was developed using a concept-based framework that combined three core elements: family planning/contraceptive use, discontinuation of contraceptive methods, and Nigeria. For each concept, relevant keywords and controlled vocabularies (e.g., MeSH terms) were identified and combined using Boolean operators. These concepts were then linked using “AND” to ensure retrieval of studies specifically addressing contraceptive discontinuation among women in Nigeria.

**Table:** 

Concept	Keywords / MeSH terms
Family planning / Contraception	“family planning”, contracept*, “birth control”, “modern contraceptive”, “long-acting reversible contraceptive”, LARC, IUD*, “injectable contraceptive”, “oral contraceptive*”, “hormonal contraceptive*”, “implant*”, “intrauterine device*”
Discontinuation / Non-use	discontinu*, “method switching”, “non-continuation”, “stopping use”, “method cessation”, “dropout”, “discontinued use”
Location	Nigeria, “Nigerian women”, “women in Nigeria”

### Inclusion criteria

This systematic review included English language quantitative observational studies published between January 2006 and December 2024 that examined contraceptive discontinuation among women of reproductive age (15–49 years). The review was conducted in 2025, and the publication period was selected to capture nearly two decades of evidence on contraceptive discontinuation while ensuring that the findings reflected contemporary FP practices and programs.

Eligible studies reported the prevalence, patterns, causes, and/or determinants of contraceptive discontinuation. Studies employing cross-sectional, cohort, or other observational designs were considered for inclusion. Experimental studies, qualitative studies, mixed-methods studies, case reports, commentaries, conference abstracts, reviews, and studies that did not report relevant outcomes on contraceptive discontinuation were excluded. The review focused on synthesizing evidence on the magnitude, trends, reasons, and statistically measurable determinants of contraceptive discontinuation among women of reproductive age.

### Data screening

This systematic review adopted a three-stage screening process for the selection of literature. Firstly, an independent search based on the search strategy outlined above was conducted, Secondly, titles and abstracts of retrieved articles were screened by the authors (AA, OI, and AA) by accessing their eligibility for inclusion in the review using the inclusion criteria. Furthermore, full texts of the selected articles were reviewed to assess their relevance to the objective of the review.

**Fig. 1:**
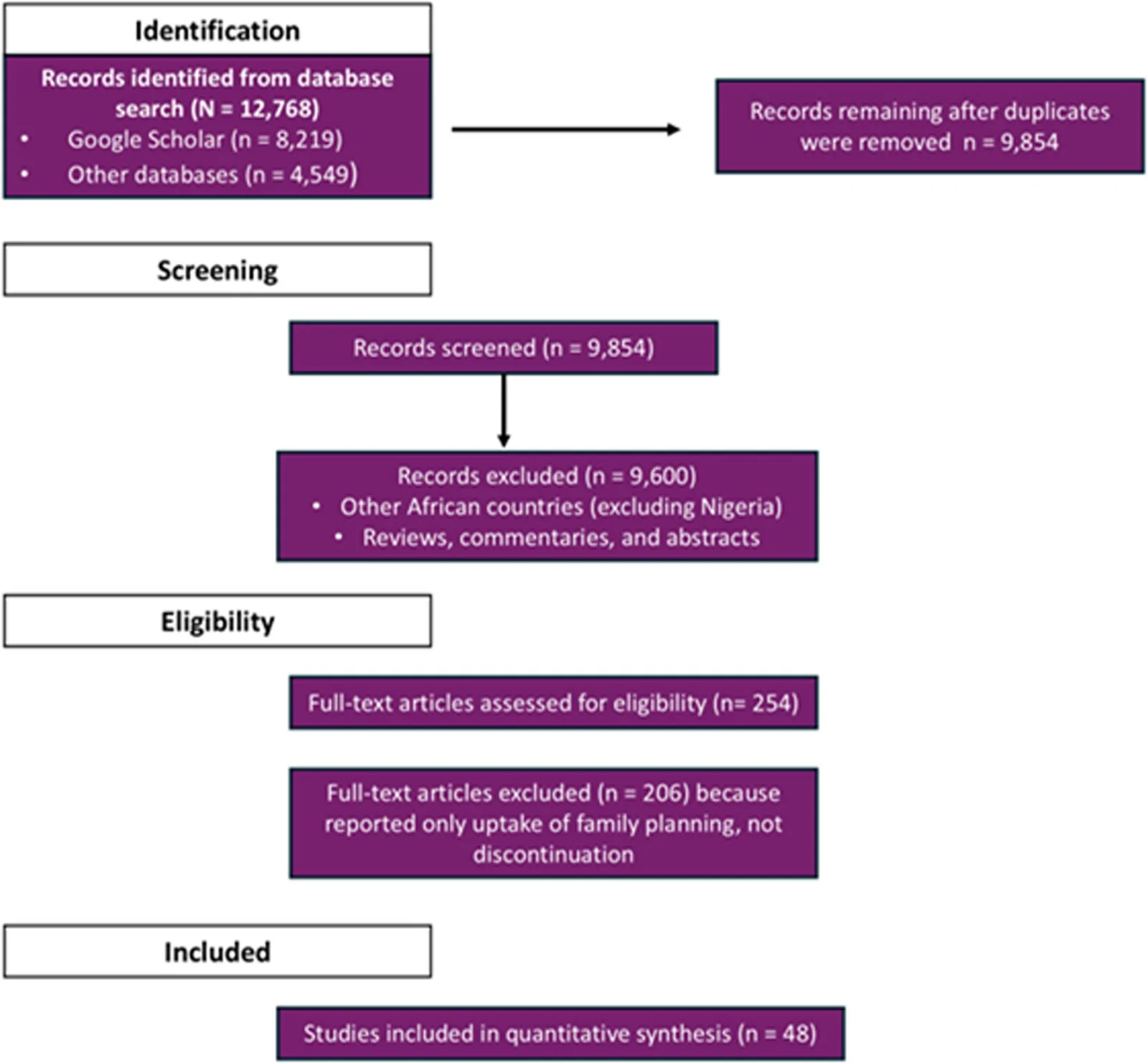
PRISMA flowchart showing the strategy adopted for screening articles

The flow chart of the search strategy is presented using the Preferred Reporting Items for Systematic Reviews and Meta-Analyses (PRISMA) framework (Fig. 1) [19]. The database search identified 12,768 records. After removal of 2,914 duplicates, 9,854 records were screened by title and abstract, resulting in the exclusion of 9,600 records. A total of 254 full-text articles were assessed for eligibility, of which 206 were excluded for not meeting the inclusion criteria. Forty-eight studies were ultimately included in the review.

The body of literature on FP discontinuation in Nigeria included both qualitative and quantitative studies, with cross-sectional surveys and Demographic and Health Survey-based analyses being the most common. A smaller number of longitudinal and mixed-methods studies also contributed nuanced insights into women’s contraceptive trajectories. Most of the retrieved research articles have been conducted in the Southwest zone of the country.

### Assessment of methodological quality

The methodological quality of the included studies was assessed using the Joanna Briggs Institute (JBI) Critical Appraisal Checklist for Analytical Cross-Sectional Studies (Table 1). Overall, the quality of evidence was moderate. Most studies clearly defined their objectives, described the study population and setting adequately, and employed appropriate quantitative study designs relevant to the research aims. Measurement of exposure and outcomes related to FP discontinuation was generally valid and reliable across studies; however, several methodological limitations were identified. Many studies did not clearly report the use of objective or standardized criteria for measuring FP discontinuation, and the identification and management of potential confounding factors were inconsistently addressed. While most studies employed appropriate statistical analyses, strategies to control for confounding, such as multivariable adjustment, were not always explicitly stated. Ethical considerations were reported in several studies, although some lacked sufficient detail. Despite these limitations, the included studies provided relevant and valuable quantitative evidence on the determinants and patterns of FP discontinuation. No study was excluded based on quality appraisal; however, methodological weaknesses were considered when interpreting the findings. Instead, identified methodological weaknesses were documented and incorporated into the interpretation of results, discussion of findings, and assessment of the strength of the evidence. This approach ensured that the review captured the full range of available evidence while acknowledging the limitations of individual studies.

**Table 1:** Quality assessment of the included articles using the JBI checklist

	Were the criteria for inclusion in the sample clearly defined?	Were the study subjects and the setting described in detail?	Was the exposure measured in a valid and reliable way?	Were objective, standard criteria used for measurement of the condition?	Were confounding factors identified?	Were strategies to deal with confounding factors stated?	Were the outcomes measured in a valid and reliable way?	Was appropriate statistical analysis used?
Oye-Adeniran et al., 2006 [20]	⎫	⎫	⎫	⎫	•	NA	⎫	⎫
Okunlola et al., 2006 [21]	⎫	⎫	⎫	⎫	•	**NA**	⎫	⎫
Adegbola and Ogedengbe, 2008 [22]	⎫	⎫	⎫	⎫	•	**NA**	⎫	⎫
Igwegbe et al., 2010 [23]	⎫	⎫	⎫	⎫	•	**NA**	⎫	⎫
Adeyemi and Adekanle, 2012 [24]	⎫	⎫	⎫	⎫	•	**NA**	⎫	⎫
Enyindah et al., 2012 [25]	⎫	⎫	⎫	⎫	•	**NA**	⎫	⎫
Ankomah et al., 2013 [26]	⎫	⎫	⎫	⎫	•	**NA**	**NA**	**NA**
Kale et al., 2013 [27]	⎫	⎫	⎫	⎫	•	**NA**	**NA**	**NA**
Balogun et al., 2014 [28]	⎫	⎫	⎫	⎫	•	**NA**	⎫	⎫
Egede et al., 2015 [29]	⎫	⎫	⎫	⎫	⎫	⎫	⎫	⎫
Lamina and Ikhile, 2015 [30]	⎫	⎫	⎫	⎫	•	**NA**	⎫	⎫
Madugu et al., 2015 [31]	⎫	⎫	⎫	⎫	⎫	⎫	⎫	⎫
Alegbeleye and John, 2016 [32]	⎫	⎫	⎫	⎫	•	**NA**	⎫	⎫
Balogun et al., 2016 [33]	⎫	⎫	⎫	⎫	⎫	⎫	⎫	⎫
Kana et al. 2016 [34]	⎫	⎫	⎫	⎫	⎫	⎫	⎫	⎫
Mobolaji et al., 2016 [35]	⎫	⎫	⎫	⎫	⎫	⎫	⎫	⎫
Njoku et al., 2016 [36]	⎫	⎫	⎫	⎫	•	**NA**	⎫	⎫
Okwudili et al., 2016 [37]	⎫	⎫	⎫	⎫	•	**NA**	⎫	⎫
Jagun, 2017 [38]	⎫	⎫	⎫	⎫	⎫	⎫	⎫	⎫
Oranu et al., 2017 [39]	⎫	⎫	⎫	⎫	•	**NA**	⎫	⎫
Ottun et al., 2017 [40]	⎫	⎫	⎫	⎫	•	**NA**	⎫	⎫
Liu et al., 2018a [41]	⎫	⎫	⎫	⎫	•	**NA**	⎫	⎫
Ezeome et al., 2018 [42]	⎫	⎫	⎫	⎫	⎫	⎫	⎫	⎫
Olamijulo et al., 2018 [43]	⎫	⎫	⎫	⎫	•	**NA**	⎫	⎫
Liu et al., 2018b [44]	⎫	⎫	⎫	⎫	⎫	⎫	⎫	⎫
Ohihoin et al., 2019 [45]	⎫	⎫	⎫	⎫	•	**NA**	⎫	⎫
Dongo et al., 2019 [46]	⎫	⎫	⎫	⎫	•	**NA**	⎫	⎫
Agida et al., 2019 [47]	⎫	⎫	⎫	⎫	•	**NA**	⎫	⎫
Alayande et al., 2019 [48]	⎫	⎫	⎫	⎫	•	**NA**	⎫	⎫
Nonye-Enyidah et al., 2020 [49]	⎫	⎫	⎫	⎫	•	**NA**	⎫	⎫
Nonye-Enyidah and Enyidah, 2020 [50]	⎫	⎫	⎫	⎫	•	**NA**	⎫	⎫
Isa et al., 2020 [51]	⎫	⎫	⎫	⎫	•	**NA**	⎫	⎫
Ahuru and Nzoputam, 2020 [52]	⎫	⎫	⎫	⎫	⎫	⎫	⎫	⎫
Bello and Agboola, 2020 [53]	⎫	⎫	⎫	⎫	•	**NA**	⎫	⎫
Awoyesuku et al., 2020 [54]	⎫	⎫	⎫	⎫	•	**NA**	⎫	⎫
Kupoluyi, 2020 [55]	⎫	⎫	⎫	⎫	•	**NA**	⎫	⎫
Ohazuruike et al., 2020 [56]	⎫	⎫	⎫	⎫	•	**NA**	⎫	⎫
Gadanya and Aliyu [57]	⎫	⎫	⎫	⎫	•	**NA**	⎫	⎫
Nonye-Enyidah et al., 2021 [58]	⎫	⎫	⎫	⎫	•	**NA**	⎫	⎫
Jemisenia et al., 2021 [59]	⎫	⎫	⎫	⎫	⎫	⎫	**NA**	**NA**
Jumbo et al., 2021 [60]	⎫	⎫	⎫	⎫	•	**NA**	⎫	⎫
Dwyer et al., 2022 [61]	⎫	⎫	⎫	⎫	•	**NA**	⎫	⎫
Agbana et al., 2022 [62]	⎫	⎫	⎫	⎫	•	**NA**	⎫	⎫
Ojoniyi et al., 2022 [63]	⎫	⎫	⎫	⎫	•	**NA**	⎫	⎫
Kupoluyi et al., 2023 [64]	⎫	⎫	⎫	⎫	•	**NA**	⎫	⎫
Cardona et al., 2024 [65]	⎫	⎫	⎫	⎫	•	**NA**	⎫	⎫
Inuwah et al., 2024 [66]	⎫	⎫	⎫	⎫	•	**NA**	⎫	⎫
Nwala et al., 2024 [67]	⎫	⎫	⎫	⎫	•	**NA**	⎫	⎫

⎫ : Yes

• : No

*NA:* Not Applicable

## Results

Across the reviewed studies (Table 2), the prevalence rates of FP discontinuation varied, ranging between 20% and 60%, with the highest prevalence found among women using short-acting methods. Early discontinuation: defined as stopping use within the first 12 months, was prevalent, especially among younger women and those with lower educational attainment. Discontinuation rates varied by method type. Condoms were discontinued primarily due to behavioral factors, such as inconsistent use, discomfort, or misconceptions, with 12-month discontinuation rates around 17–18%. Oral contraceptives were discontinued within the first 12 months by 21–59% of users, largely due to side effects, daily regimen demands, and personal fertility intentions. Injectables showed discontinuation rates of 19.8–53.3%, mostly within the first 3–6 months, driven by side effects and the desire for pregnancy. Long-acting reversible contraceptives (LARCs), including implants and IUDs, had lower discontinuation rates (7% – 42.9% at 12 months), though early discontinuation was associated with menstrual irregularities, desire for pregnancy, or method-specific side effects.

**Table 2:** Summary of articles included in the systematic review

Author	Objective	Study design	Contraceptive method	Sample size	Results
Oye-Adeniran et al., 2006 [20]	To assess possible reasons for contraceptive non-use despite reported high awareness in Nigeria	Secondary data obtained from the 203 Nigeria Demographic Health Survey	Not stated	2,001 women of reproductive age	The reasons for discontinuation were desire for more children (61.7%), did not expect to have sex (17.9%), and fear of side effects (17.2%). The reasons for discontinuation of the methods were similar in both urban and rural areas. Also, 54.6% of women who have discontinued family planning (FP) had the intention to use it in the future.
Okunlola et al., 2006 [21]	To determine the discontinuation rate among IUCD users at the University College Hospital, Ibadan, Nigeria	A 5-year retrospective analytical study of the records of patients using an IUCD seen at the FP clinic between 1 January 1998 and 31 December 2003.	10-year intrauterine device (IUD)	867 women	The prevalence of 10-year IUD discontinuation over 5 years was 29.8%, with the highest rate at 1 year (10.1%) and least after 5 years (2.8%).
Adegbola and Ogedengbe, 2008 [22]	To determine the characteristics of women using the IUCD, the main reasons for acceptance, complications arising from usage and discontinuation rate as well as the reasons for discontinuing	Retrospective study of the case records of 2,574 new IUCD acceptors attending the Department of Obstetrics and Gynaecology FP clinic of the Lagos University Teaching Hospital between 1990 and 1994	Not stated	-	Mean age: 31.3 ± 5.5 years, and the mean number of children alive was 3.6 ± 1.8. By 12 months, the discontinuation rate was 13.9%, 26.3% at 24 months, while the cumulative discontinuation rate was 47% at forty-eight months. Commonest reasons for discontinuation was the desire to get pregnant (26.6%) and menstrual disorders (6.7%)
Igwegbe et al., 2010 [23]	To determine the 10-year clinical experience with Cu-T IUD at the Nnamdi Azikiwe University Teaching Hospital, Nnewi, Nigeria	10-year retrospective study	10-year IUD	861 hormonal IUD acceptors out of the total population of 1,345 FP acceptors	Overall prevalence of discontinuation: 55.1% Discontinuation was largely influenced by women/spousal desire for pregnancy/another childbirth
Adeyemi and Adekanle., 2012 [24]	Secondary data of clients using progestogen-only injectable contraceptive between 1st January, 2001 and 31st December, 2009 at the Ladoke Akintola University of Technology Teaching Hospital, Osogbo, Nigeria	Secondary data retrieved from clients’ case notes	Not stated	433 women of reproductive age	The mean age of the women was 34.0 ± 5.7 years, and the average parity was 3.5 ± 1.5. In all, 22.4% women had discontinued contraceptives, out of which 81.9% cited menstrual abnormality as the commonest reason for the discontinuation. Other reasons for discontinuation were excessive weight gain (6.0%), desire for pregnancy (4.8%), attainment of menopausal age (3.6%), nipple pain (2.4%), and inability to keep appointment (1.2%). Women with lower parity had significantly (*p* < 0.001) lower duration of use than those of higher parity. Also, women who had stillbirth as antecedent delivery significantly (*p* < 0.01) discontinued the method earlier than those who had a live baby. Women who used the method to limit their families significantly (*P* < 0.01) stayed longer than those using the method for child spacing. Those who had no complaint significantly (*p* < 0.05) stayed longer on the method than those with complaints.
Enyindah et al., 2012 [25]	To determine the uptake rate as well as the effectiveness, side effects and reasons for discontinuation of 10-year IUD at the University of Port Harcourt Teaching Hospital, Port-Harcourt, Nigeria between 1st January 1997 and 31st December 2006	Secondary data obtained from clients’ case notes	10-year IUD	1,632 women who took the 10-year IUD FP method	The mean was 33.5 ± 4.6 years, while the mean parity was 3.8 ± 2.6. The mean duration of 10-year IUD use was 20.8 ± 2.4 months. Prevalence of IUD discontinuation: 42.9%. Th e common reasons for discontinuation were desire to get pregnant (42.0%), menorrhagia (15.2%), desire to switch 10-year IUD to another method (6.5%) and vaginal discharge (6.2%).
Ankomah et al., 2013 [26]	To explore the key cultural and societal perceptions, beliefs and practices that impede the uptake of contraceptives among young married Nigerians.	Qualitative interviews conducted through 13 focus group discussions conducted across the six geopolitical zones in Nigeria	Not stated	-	For some women, it was not the mother-in-law but their own mothers who were asking them to discontinue the use of contraceptives. The mothers think that having more children ensures marital stability
Kale et al., 2013 [27]	To evaluate the perception of nurse-midwives on the factors influencing oral contraceptive use, misuse, non-use and discontinuation among clients in Ibadan, Nigeria.	A qualitative randomized survey data of 88 nurse-midwives who have experience with clients on oral contraceptives	Not stated	-	Mean age of participants: 32 ± 0.1 years The major causes of contraceptive discontinuation were fear of side effects (59.1%), and the perception that oral contraceptives can make clients fall ill (30.7%). Significant association existed between nurse-midwives’ perception and factors influencing oral contraceptive use.
Balogun et al., 2014 [28]	To determine the safety, efficacy and acceptor characteristics of Implanon at the family planning clinic of University of Ilorin Teaching Hospital, Ilorin, Nigeria.	A retrospective review of 88 clients who used Implanon from January 2007 to December 2011 at the family planning clinic	Not stated	88 clients who had used Implanon	The mean age of Implanon users in the study was 33.4 ± years. Discontinuation rate was 26.1%. The commonest reasons for discontinuation were the desire to get pregnant (35%), menstrual irregularities (26.1%), and undesirable weight gain (8.7%).
Egede et al., 2015 [29]	To determine the prevalence and factors influencing the choice and sources of contraceptive options among 330 market women of reproductive age in Ebonyi State, Nigeria.	Descriptive cross-sectional	Not stated	-	Prevalence of FP discontinuation: Not stated. Major reasons for discontinuation of a contraceptive method were: i. The desire for more children (11.2%) ii. Spousal disapproval (9.1%) iii. Side effects 7.3%) iv. Religious beliefs (2.1%) v. Cultural beliefs (0.6%). The most common reason for not using any form of contraception
Lamina and Ikhile, 2015 [30]	To examine the socio-demographic characteristics, main source of information about family planning and contraceptive choices of 733 new clients attending the family planning clinic of Olabisi Onabanjo University Teaching Hospital, Sagamu, Nigeria between 1st January 2006 and 31st December 2011.	A retrospective descriptive analysis of the case records of all new clients	10-year IUD	-	Two (13.3%) of the 15 clients with evidence of discontinuation before one year got pregnant while on the contraceptive method and others stopped due to the side-effects which included menorrhagia, chronic backache, and disturbance of sexual activity by IUD tag.
Madugu et al., 2015 [31]	To describe the experience on the use of Jadelle/Implanon contraceptives that was recently introduced into Ahmadu Bello University Teaching Hospital, Zaria, Nigeria.	A retrospective study of clients that accepted Jadelle/ Implanon from June 2009 to November 2013 at Ahmadu Bello University Teaching Hospital, Zaria, Nigeria.	Implant (Jadelle/ Implanon)	798 clients	The discontinuation rate was 26% for Jadelle and 19% for Implanon. Menstrual problems and desire for pregnancy were the commonest reasons for discontinuation of Jadelle (64.8% vs. 11.2%) and Implanon (68.7% vs. 0.3%). These were also the major side effects experienced for each method.
Alegbeleye and John, 2016 [32]	To determine the prevalence and pattern of contraceptive use, and to identify factors influencing use of modern contraceptive methods	A retrospective review	Modern FP method	1,060 new FP clients	The mean age of clients was 34.22 ± 5.42 years while the mean parity was 3.51 ± 1.47. The contraceptive discontinuation rate was 13.8%, and it was mainly due to the desire for another pregnancy (39.7%). Age, parity and marital status of the clients were significantly associated with discontinuation of a method (*p* < 0.05). The contraceptive prevalence rate markedly increased over the five-year period.
Balogun et al., 2016 [33]	To assess the role of male partner’s support on spousal modern contraception at the family planning clinic of the University of Ilorin Teaching Hospital, Nigeria, between December 2013 and April 2014.	Prospective cross-sectional survey	Not stated	305 women of reproductive age	Previous methods were discontinued due to desire for pregnancy (28.7%), complications (27.1%), male partner hindrances (23.3%), peer influence (11.6%), or other personal reasons (9.3%).
Kana et al. 2016 [34]	To assess the prevalence and identify determinants of contraceptive use in Gumau, a rural community of Bauchi State, Northeastern Nigeria.	Quantitative cross-sectional study	Oral contraceptives	Not stated	Daily ingestion of oral contraceptives and lack of husband’s support were major reasons for discontinuation of contraceptives use in the past.
Mobolaji et al., 2016 [35]	To explore the ethnic variations in contraceptive discontinuation among married women of childbearing aged 15–49 years	Descriptive cross-sectional	Not stated	5,992 women	Discontinuation was significantly higher among Hausa/Fulani (40.2%) and the ethnic minority groups (37.8%) compared to Yoruba (33.6%) and Igbo (35.6%) women (*p* < 0.05). Determinants of FP discontinuation were number of living children, age at first marriage, and desire for more children (*p* < 0.05). Top reasons for discontinuation were infrequent sex (18.1%), menopausal effects/hysterectomy (17.8%), fear of side effects (17.5%), and respondent’s opposition (10.1%).
Njoku et al., 2016 [36]	To determine the profile of the acceptors, side effects and indication for discontinuation of progestogen-only injectable contraceptives (POIC) at the University of Calabar Teaching Hospital, Calabar	A retrospective study of clients who used POIC at the family planning unit between 1st January 2008 and 31st December 2014	POIC	1,392 women	Age range: 17–46 years Prevalence of discontinuation of POIC: 19.8%. Major reasons for discontinuation included secondary amenorrhea (48.1%), desire to get pregnant (31.4%), weight gain (9.8%), spotting (7.8%), and hypertension (7.8%). POIC was associated with multiple side-effects, such as secondary amenorrhea (47.7%), intermenstrual spotting (22.8%), and weight gain (12.5%).
Okwudili et al., 2016 [37]	To re- evaluate the use, effectiveness, acceptability and side effects of depot medroxyprogesterone acetate in Port Harcourt, Southern Nigeria	Review of case files of clients who received used depot medroxyprogesterone acetate at the FP clinic, University of Port-Harcourt Teaching Hospital, (UPTH), Port Harcourt Nigeria, between 1st January 2004 and 31st December 2013.	Depot Medroxyprogesterone Acetate	677 women out of the total of 7001 new acceptors	Prevalence of discontinuation of depot medroxyprogesterone acetate: 22.5%
Jagun, 2017 [38]	To determine the family planning practices among parturients and assess the factors influencing the uptake of contraceptives in the semi urban and rural population attending antenatal clinics in Ijebu-ode, Nigeria between August – November 2015.	Cross-sectional quantitative survey	Not stated	131 women of reproductive age	The age range of the respondents was 18-42years, while parity ranged from 1–5. Overall, 40.3% women had discontinued FP, largely due to desire for contraception (61.5%), undesirable weight gain (15.4%), irregular periods (15.8%), amenorrhea (6.2%), menorrhagia (4.6%) and difficulty in accessing the method (1.5%).
Oranu et al., 2017 [39]	To determine the associated factors in the declining acceptance and growing discontinuation of POIC at the University of Port-Harcourt Teaching Hospital.	A retrospective study of the case notes of clients who accepted and used POIC at the family planning clinic of the University of Port Harcourt Teaching Hospital between 1st January 2004 and 31st December 2013	POIC	1,075 women	Prevalence of discontinuation of POIC: 84.9%. Secondary amenorrhea and weight gain, 72.7% and 14.7% respectively, were the main side effects noted by clients to discontinue the FP method. Method switch occurred among 21.6%.
Ottun et al., 2017 [40]	To evaluate the choice and trend of contraceptive usage over 3 years, the most preferred method and the adverse effects associated with each type of contraceptive method among the clients attending the family planning clinic at Lagos State University Teaching Hospital, Nigeria	Retrospective study of 1,843 clients attending the FP clinic at LASUTH from January 2011 to December 2013	10-year IUD, Injectables, Implants, and Oral contraceptive pills	-	Mean age: 34.1 + 5.4 years. Discontinuation rates were 26.4% in 10-year IUD users, 17.8% of combined injectable/implants users, 23% among oral contraceptive pills and 17.9% among those using barrier methods over one year period.
Liu et al., 2018a [41]	To examine the sociodemographic predictors of continued DMPA-SC use after 3 months and characterize the additional influences of contraceptive counselling quality and experiences of side effects on continuation in Ekiti, Kwara, Lagos, Ogun, Ondo, Osun, and Oyo States in Nigeria.	Prospective follow-up study; Convenience sampling, phone interviews	DMPA-SC	541 DMPA-SC users	The continuation rate for DMPA-SC was 49.7%. Women with some college education or more (OR = 2.8; 95% CI: 1.1–7.1), and those with four or more children (OR = 2.9; 95% CI: 1.1–7.7) were more likely than their less educated and lower parity counterparts to obtain another dose (OR = 2.0; 95% CI: 1.1–3.5). Experiencing more bleeding reduced the likelihood of DMPA-SC continuation at 3 months (OR = 0.2; 95% CI: 0.2–1.2).
Ezeome et al., 2018 [42]	To assess the factors influencing the use of the Creighton model system of natural family planning and its effectiveness in a subpopulation of women in Enugu, Southeast Nigeria	A cross-sectional descriptive study of 45 couples attending the family planning clinic of a private hospital in Enugu, Nigeria	Not stated	-	Age range of the women was 22–47 years and a mean of 32 years, with a mean parity of 4. The prevalence of FP discontinuation: 15.6%. The major reasons for discontinuation are tension between couples during fertility and uncertainty about fertile period.
Olamijulo et al., 2018 [43]	To determine the pattern of use of 10-year IUD, sources of referral, its associated complications and reasons for its discontinuation over a 10‑year period at the family planning clinic of the Lagos University Teaching Hospital, Nigeria.	Secondary data of FP clients over a 10‑year period (January 1, 2006, to December 31, 2015) retrieved from case notes	10-year IUD	1,726 women of reproductive age	Overall, 40.7% of the women discontinued the use of the 10-year IUD one year after onset of use. The most common reason for discontinuing the use of IUD was the occurrence of undesirable effect (44.8%) such as pain, abnormal vaginal discharge, and menstrual disorders. Also, 43% women discontinued 10-year IUD due to the desire for pregnancy.
Liu et al., 2018b [44]	To determine the sociodemographic predictors of continued DMPA-SC use after 3 months, and additional influences of contraceptive counselling quality and experiences of side effects on continuation.	Descriptive cross-sectional study of 311 DMPA-SC users in Nigeria	DMPA-SC	311 DMPA-SC users followed up at 3 months	Prevalence of DMPA-SC discontinuation: 50.3% Prevalence of DMPA-SC continuation: 49.7%. Sociodemographic predictors of continued DMPA-SC use at 3 months were: having college or higher education (OR = 2.8; 95% CI: 1.1–7.1), and parity ≥ 4 (OR = 2.9; 95% CI: 1.0-7.7). Higher quality during the initial counseling session increases the likelihood of getting another dose of DMPA-SC (OR = 2.0; 95% CI: 1.1–3.5) while bleeding reduced the likelihood of continuation after 3 months (OR = 0.2; 95% CI: 0.1–0.3).
Ohihoin et al., 2018 [45]	To determine the use and pattern of contraceptive uptake among HIV‑infected clients registered for antiretroviral therapy services at an HIV outpatient clinic in South‑West Nigeria; factors influencing contraceptive uptake among HIV‑infected clients; and the contraceptive failure and/or discontinuation rate	Review of the database of HIV-positive clients on antiretroviral therapy	3-months injectables and condoms	537 men and women	Prevalence of FP discontinuation: 51%. Reasons for discontinuation include desired conception in 54 (16.6%), menstrual irregularities in 45 (13.8%), and spouse disapproval in 11 clients (2%).
Dogo et al., 2019 [46]	To determine the sustainability of FP in the context of the 2004 population policy in the Federal Capital Territory (FCT) of Nigeria	Primary data collection involving the administration of questionnaires to 1,062 household heads	Not stated	-	Overall prevalence of FP discontinuation: 31.7%. Major reasons for discontinuation were the desire for more children (45.0%), fear of side effects (12.2%), lack of access/cost (10.2%), spousal opposition (8.8%), religious consideration (8.7%), and marital challenge (7.0%). Only occupation (*r* = 0.115) of respondents was recognised to bring about noteworthy changes in people’s desire to practice family planning in the future.
Agida et al., 2019 [47]	To determine the pattern of contraceptive method-mix among adopters	5-year retrospective review of FP adopters attending the FP clinic of the University of Abuja Teaching Hospital, Abuja, Nigeria	Implants (Jadelle or Implanon)	1,620 FP adopters	Mean age: 31.8 ± 6.0 years. Overall, 588 (36.6%) women accepted Implants (Jadelle or Implanon), while 520 (32.1%) accepted intrauterine contraceptive device, and 391 (24.1%) accepted injectable contraceptives (Depo-Provera and Noristerat). Prevalence of discontinuation within 1 year of use: 8.9% Prevalence of discontinuation within 5 years: 21.2%
Alayande et al., 2019 [48]	To determine the profile of FP discontinued users and the prevalence of FP commodity discontinuation among clients in the three high volume secondary health facilities of Kaduna metropolis.	9134 FP client cards for the three years were reviewed for evidence of FP switching or discontinuation out of which 809 client cards were found with such evidence.	Not stated	-	The average age of discontinued users was 30 years. The commonest reason for discontinuation of contraceptives was health concerns or side effects.
Nonye-Enyidah et al., 2020 [49]	To determine the prevalence rate, side effects, discontinuation rate and indications for discontinuation of DMPA at Rivers State University Teaching Hospital (RSUTH), Port Harcourt	A retrospective study of 874 clients attending the FP clinic at the RSUTH from 2015 to 2019	DMPA	-	Age range was 19–47 years, and the modal parity was 2. The prevalence of DMPA use was 11.6%, while the discontinuation rate was 32.7%. The commonest reasons for discontinuation were secondary amenorrhoea (24.2%), irregular vaginal bleeding (24.2%), desire to get pregnant (18.1%), and menorrhagia (12.1%).
Nonye-Enyidah and Enyidah, 2020 [50]	To determine and compare the prevalence rates, side effects, discontinuation rates and indications for discontinuation of two subdermal implants (Jadelle and Implanon) at Rivers State University Teaching Hospital (RSUTH), Port Harcourt, Nigeria.	Secondary data retrieved from case files of 874 clients attending the FP clinic at the Rivers State University Teaching from 1st January 2015–31st December, 2019	Implant and Jadelle	-	Prevalence of FP discontinuation: 26.4% (Implanon – 65.2% vs. Jadelle – 34.8%). Menstrual abnormality (24.1%), desire for pregnancy (46.4%), irregular vaginal bleeding (8.9%), and weight gain were the commonest reasons for discontinuation of subdermal implants (Jadelle and Implanon)
Isa et al., 2020 [51]	To determine the trends, uptake, and reasons for discontinuing long-acting reversible contraception in University of Maiduguri Teaching Hospital, Maiduguri	5-years retrospective study (January 2015 – December 2019)		4,064 adopters of any LARC method	Reason for discontinuation included the desire for more children, irregular menstrual flow, contraceptive faire; husband refusal, weigh gain, infection and perception that the IUD string is missing. Clients who had no formal education were more likely to discontinue due to desire for more children (39.1%), menstrual irregularity (26.6%) compared to those who had tertiary education (32.1%). Unemployed women were more likely to discontinue LARC due to desire for more children (38.0%) while civil servants were more likely to continue using LARC till the time elapsed (26.5%).
Ahuru and Nzoputam, 2020 [52]	To assess the coverage, barriers to usage and predictors of modern contraception among women of reproductive age in Ughelli North Local Government Area (LGA), Delta State, Nigeria.	Community-based cross-sectional household survey	Injectables, Oral contraceptive pills, Implants, Male condoms, Female condoms, and traditional FP methods.	384 women of reproductive age (married or in a consensual union) but having no intention to give birth in the next two to three years.	Pattern of contraceptive use: Injectables (26.2%), Oral pills (24.6%), Implant (14.8%), Male condoms (8.2%), bilateral tubal ligation (3.3%), female condom (1.6%), and traditional methods (21.3%). Discontinuation rate for modern contraceptive: 31.8%. Motivators for discontinuing contraceptive use: Fear of side effects (39.6%), desire for childbearing (16.7%), husband’s desire to stop contraceptive use (6.3%), and high costs of contraceptives (4.2%).
Bello and Agboola, 2020 [53]	To determine the reasons for IUD discontinuation among women of reproductive age who visited the FP clinic of the University College Hospital, Ibadan, Nigeria	Secondary data	10-year IUD	12,019 women	About two-fifth (4085) of them had IUD insertion while 1,961 had removal of IUD. The acceptance and discontinuation rates were 40.1% and 48.0% respectively. The mean age of women who had IUD removal was 37.8 ± 8.6 years and majority 528 (26.9%) were between 30 to 34 years. The mean duration of IUD use was 6.2 ± 4.6 years. The total discontinuation rates were 20.3% at 12 months, 25.6% at 24 months and 32.7% at 36 months. The commonest reasons for IUD removal were side effects with 27.6% comprising of heavy menstrual bleeding, lower abdominal pain, recurrent vaginal infections, and inability to feel strings. Other reasons included clients’ preference (27.3%), desire to conceive (21.4%), expired device (12.7%), and menopause (10.8%). A significant relationship exists between client’s preference for discontinuation of IUD and age (*p* < 0.001) but their educational status (*p* = 0.306) and parity (*p* = 0.90) were not associated with IUD discontinuation.
Awoyesuku et al., 2020 [54]	To determine the discontinuation rates, reasons for discontinuation or switching, and assess associated factors	Retrospective cross-sectional of women 15–48 years who accepted a modern method, between January 2014 to December 2018 at the University of Port-Harcourt Teaching Hospital, Nigeria	Injectables, 10-year IUD, and Implants	349 women	Mean age was 32.0 ± 5.6 years and median parity was 3. All-method discontinuation rate was 29.0% at 12 months and 31.5% by 24 months. Method specific discontinuation at 12 months were 53.3% for injectables, 38.2% for 10-year IUD, and 23.8% for implants, and the difference was significant (*p* = 0.018). Reasons for discontinuation were reduced need for FP (64.5%), side effects (26.8%) and expiration (7.2%).
Kupoluyi, 2020 [55]	To examine intimate partner violence (IPV) as a factor affecting contraceptive discontinuation in Nigeria	Secondary data analysis of 2013 NDHS women dataset	Not stated	1341 women in a union in the domestic violence module, who have experienced IPV, and are using any contraceptive and are not sterilized in the 12 months periods	Prevalence of contraceptive discontinuation among survivors of IPV: 20.1% Women who have experienced any type of IPV are 1.28 times more likely to have discontinued contraceptive use although they are still at risk of becoming pregnant (AOR = 1.28, CI: 1.15–1.91; *p* < 0.05) than those who have not experienced IPV. The tertiary level of education (AOR = 3.94, CI = 1.67–9.29; *p* < 0.05), unemployed status (AOR = 1.97, CI = 1.07–3.62; *p* < 0.05), and higher marital duration of 20 years and above (AOR = 4.89, CI = 2.26–10.57; *p* < 0.05) significantly influenced women who have experienced any types of IPV to discontinue contraceptives.
Ohazuruike et al., 2020 [56]	To assess the predictors of discontinuation of FP among women in Nigeria	Retrospective cohort study of FP acceptors at the FP unit of the College of Medicine, Lagos University Teaching Hospital, Lagos, Nigeria between October 2007 and September 2010	5-year Jadelle Implants	517 consecutive new Jadelle acceptors	The mean age of Jadelle acceptors was 32.9 (S.D: ±5.4) years and uptake rate of Jadelle was 61.8%. The overall discontinuation rate was 19%, while the 1-year, 2-year and 3-year Jadelle discontinuation rates were 7.1%; 27.0% and 58.1% respectively. Increased age, (*p* = 0.047) and previous contraceptive use (*p* < 0.001) were independent predictors of Jadelle discontinuation. Menstrual irregularity (51.4%) and intention to get pregnant (40.3%) were the commonest reasons for discontinuation.
Gadanya and Aliyu, 2021 [57]	To assess contraceptive discontinuation among women of reproductive age	A cross-sectional study designed with concurrent mixed methods of data collection	Pills, Injectables, and IUD	350 women, out of which 168 had discontinued a contraceptive method	Prevalence of FP discontinuation: 48% Method-specific discontinuation rates were: 35.1% for Implants, 33.9% for Injectables, 21.4% for Pills, and 15.5% for IUD. Most common reasons for discontinuation were as follows: Experiences of side effects (67.1%), intention to get pregnant (59.5%), method failure (16.7%), method switch (12.0%), and husband’s disapproval (9.5%)
Nonye-Enyidah et al., 2021 [58]	To determine the pattern of previous contraceptive usage, uptake rates of the contraceptives, the continuation and discontinuation rates and the sources of prior contraceptives.	A retrospective study of the records of clients’ attending the FP clinic at the Rivers State University Teaching Hospital between January 2015 and December 2019.	Not stated	874 women	Age range for the study was 19-51years. Prevalence of discontinuation of FP: 35.2%
Jemisenia et al., 2021 [59]	To examine the process of condom discontinuation among members of the National Youth Service Corps in Nigeria	Qualitative study of young Corps members in Enugu	Male condoms	80 young male and female Corps members with ages between 23 and 30 years	The motivations for condoms discontinuation included: i. Physical discomfort and decreased sensation experienced during intercourse (55.2%) ii. Feelings of pain associated with condoms use (60% among women, and 50.3% among men) iii. Misconception that condom use could lead to cancer in women There was no gender difference regarding the intermittent condom discontinuation behavior.
Jumbo et al., 2021 [60]	To determine the uptake of long-acting reversible contraception and assess the characteristics of acceptors of these methods in the area	Retrospective study of clients’ who visited the FP unit of the University of Abuja Teaching Hospital over a 5-year period, from 01 January 2015 to 31 December 2019	Not stated	-	Prevalence of FP discontinuation: 23.1%, mainly due to the desire to conceive or switch FP methods. The commonest complications of LARC were spotting and menorrhagia
Dwyer et al., 2022 [61]	To assess women’s contraceptive continuation nine months after acceptance of contraceptive services from community pharmacists and patent and proprietary medicine vendors in Kaduna and Lagos States	Prospective longitudinal survey of contraceptive users	All contraceptive methods	491 women who received a contraceptive method at nine months after receiving an FP service	Prevalence of contraceptive discontinuation: 11%
Agbana et al., 2023 [62]	To examine the predictors of FP method discontinuation among sexually active Nigerian women.	Secondary data (NDHS) analysis between 2013–2017	Male condoms, emergency contraceptive pills, non-emergency contraceptive pills, injectables, Implants, and IUD	4,553 women aged 15–49 years who had stopped using FP methods	Prevalence of modern FP discontinuation: 71.1%. Top reasons for FP discontinuation included: the desire to become pregnant (36%), fear of adverse effects (15%), method failure/pregnancy (13%), infrequent sex/husband’s absence (11%), and the desire for a more effective method (9%). Discontinuation was higher for LARCs than short-term methods. Predictors of modern FP discontinuation were secondary education (OR = 1.3, 95% CI: 1.0-1.7), Islamic religion (OR = 1.3, 95% CI: 1.1–1.6), residence in the South-East geopolitical zone (OR = 0.2, 95% CI: 0.2–0.3), having three or more children (OR = 0.8, 95% CI: 0.7-1.0) and having paid employment (OR = 0.8, 95% CI: 0.7-1.0).
Ojoniyi et al., 2022 [63]	To identify method of contraceptive, and reasons for discontinuation of contraceptives among unmarried adolescents aged 15–19 years in Nigeria.	Analysis of secondary data (2018 NDHS)	Not stated	324 never married adolescent girls who had ever used a method to prevent pregnancy	Prevalence of contraceptive discontinuation: 20% Most unmarried adolescents who reported ever discontinuing a contraceptive method were older, have completed secondary education, reside in urban areas, were at least 15 years at sexual debut, and were from richer household. Most reported reasons for discontinuation were Infrequent sex and inconvenience in use.
Kupoluyi et al., 2023 [64]	To examine the prevalence and associated factors of modern contraceptive method discontinuation among sexually active married women in Nigeria.	Analysis of 2018 NDHS	Injections, Implants/ Norplant, lactational amenorrhea, male condoms, pills, and IUD	3,353 currently sexually active married or in union women who have ever used a modern contraceptive 5 years before the survey and with complete reproductive histories and are not sterilised or declared infecund	Pattern of discontinuing contraceptives: injectables (25.2%), Implants/Norplant (22.4%), Lactational amenorrhea (16.4%), Male condoms (15.7%), Pills (12.8%), and IUD (6.0%). The commonest reasons for modern contraceptive discontinuation were the need to become pregnant (36.1%), followed by the need for a more effective method (19.1%), side effects/health concerns (14.1%), and became pregnant while using (13.4%). Associated factors of modern contraceptive discontinuation among sexually active married women in Nigeria were: marital duration (aOR = 3.0; 95%CI: 1.5–6.2), visitation to a health facility in the last 12 months before the survey (aOR = 0.6; 95%CI: 0.4–0.8), education (aOR = 2.0; 95%CI: 1.2–3.4) and region of residence (aOR = 2.7; 95%CI: 1.6–4.7).
Cardona et al., 2024 [65]	To determine whether the fulfilment of contraceptive preferences affected womens’ decisions to continue, switch, or stop using contraception.	Secondary data from 1,150 women in the Measurement, Learning & Evaluation project implemented for over 4 years in 11 cities across Senegal, Kenya, and Nigeria	Not stated	-	Prevalence of FP discontinuation: 40.9% The percentage of women who continued using the same method of contraception was higher for women with fulfilled contraceptive preferences than for women with unfulfilled preferences – 60.0% compared to 44.5%, respectively. Women with fulfilled contraceptive method preferences registered lower rates of switching and discontinuation than women with unfulfilled preferences – 16.0% and 23.2% for switching and 24.0% and 32.3% for discontinuation, respectively.
Inuwah et al., 2024 [66]	To determine the trend of modern contraceptive use and the reasons for their discontinuation among women of reproductive age accessing the FP clinic in Modibbo Adama University Teaching Hospital Yola, Adamawa state.	Retrospective descriptive studies of all the new contraceptive acceptors over a period of 6 years (2014–2019 inclusive)	Not stated	-	Prevalence of FP discontinuation: 17.7% Age, parity and marital status were significantly associated with discontinuation of any modern contraceptive methods (*p* < 0.001) Menstrual irregularities (29.1%), desire for pregnancy (25.6%), lower abdominal/back pain (15.2%), infections (9.2%) were the most cited reasons for discontinuation, while displacement of the IUD rod (6.5%), weight changes (5.2%), and method failure (2.3%) were the least cited reasons for discontinuation
Nwala et al., 2024 [67]	To examine continuation rate and satisfaction with hormonal IUD at three and 12 months	Prospective longitudinal phone survey of users of the hormonal IUD	Hormonal IUD	208 users of the hormonal IUD FP method at two weeks post-insertion	The prevalence of hormonal IUD discontinuation was 3.1% at three months post-insertion, and 8.2% at 12 months post-insertion. There was no significant association between FP discontinuation and age, fertility intention, or counseling.

The reasons for discontinuation were diverse and often multifactorial. The desire to become pregnant was one of the most cited motivations, particularly among women who had achieved their desired birth spacing interval. Side effects such as irregular or heavy bleeding, weight gain, or amenorrhea, were also widely reported and strongly associated with hormonal contraceptive methods. These physiological responses often triggered concern, especially in contexts where fertility is closely tied to womanhood and marital expectations. Additional reasons included spousal disapproval, where lack of partner support or outright opposition led to method cessation. This was particularly evident in patriarchal settings or among women who initiated contraceptive use without prior discussion with their partner. Religious beliefs and cultural norms also played a role, with some users discontinuing due to internalized or community-driven perceptions that contraceptive use was immoral or unnatural. Finally, misinformation and fear of long-term health consequences, including infertility and cancer, were prominent, particularly among young or first-time users (Table 2).

## Discussion

This systematic review provided a comprehensive synthesis of evidence on contraceptive discontinuation in Nigeria, revealing significant patterns in the prevalence, methods discontinued, and underlying reasons. The findings reinforce existing concerns about discontinuation as a critical gap in contraceptive uptake and its sustained use in Nigeria.

Across the reviewed studies, the prevalence of contraceptive discontinuation varied widely, from 20% to 60%, depending on study location, population subgroup, and the timeframe used to define discontinuation. These figures are consistent with national-level data from the 2018 and 2023–2024 Nigeria DHS, which reported a 12-month discontinuation rate of 35% and 38% respectively for modern FP methods [17, 68]. Similar patterns have been observed across sub-Saharan Africa [1, 3]. For instance, in Ethiopia, the 12-month discontinuation rate for all modern methods was 37% [69], while in Kenya it stood at 31% [70]. These rates remain higher than those reported in many high-income countries such as the United States, where method-related discontinuation rates tend to be below 20% [71]. These differences may reflect differences in service quality, method mix availability, health system support, and sociocultural context.

Injectables and oral pills were identified in this review as the most discontinued methods in Nigeria. This aligns with findings from Ghana, Uganda, and Mozambique, where injectables also dominate method choice and discontinuation patterns due to side effects, the need for resupply, and limited long-term protection [72–74]. In contrast, in countries with greater uptake of LARCs such as the IUD or implants, like Bangladesh and Vietnam, discontinuation rates are typically lower and more delayed, often reflecting differences in counseling quality and provider training [75, 76].

Method-related concerns, such as heavy bleeding, emerged as one of the leading reasons for discontinuation in the reviewed studies. This is consistent with other Nigerian studies out of the scope of this review, such as those conducted in Lagos and Kaduna, where fear of side effects like menstrual irregularities and weight gain were prominent discontinuation drivers [20, 77]. These findings also mirror regional evidence from Burkina Faso, where nearly half of women who discontinued contraceptives did so due to side effects [78]. Globally, side effects are the most frequently cited reason for discontinuation, regardless of method or geography, highlighting the universal need for accurate, anticipatory counseling [79].

Another recurrent reason for contraceptive discontinuation reported in this review was fertility-related motivations, including the desire to conceive. This reason was commonly cited across the studies included in this review just as it has been documented in Tanzania and Malawi, where many women use contraception for spacing rather than limiting births [80, 81]. This is a critical distinction in fertility intentions that has implications for discontinuation: in high-fertility contexts like Nigeria, where childbearing remains highly valued, discontinuation may not always reflect dissatisfaction but rather fulfilled reproductive goals.

From this review, spousal opposition and limited partner communication were also cited as significant barriers to continued contraceptive use. Several studies in this review identified male disapproval as a determinant of discontinuation, echoing findings from northern Nigeria and other patriarchal settings in other developing countries [82–84]. In contrast, countries where male involvement is integrated into reproductive health programming, such as Rwanda, have reported lower discontinuation rates and improved contraceptive continuity [85]. This suggests that gender norms and relationship dynamics substantially shape contraceptive trajectories and may either support or undermine sustained use.

The review further revealed systemic factors, particularly poor-quality counseling and limited method choice, as contributors to premature discontinuation. Women who reported inadequate counseling were more likely to discontinue without switching to another method. This is in line with evidence from the Philippines and India, where discontinuation was significantly associated with low-quality service delivery and limited follow-up [86, 87]. In Nigeria, the skewed contraceptive method mix, with over-reliance on injectables and pills, may itself be a function of weak health system integration of LARCs and user-centered care models [88].

Interestingly, although the reviewed studies varied in design, few employed longitudinal methodologies that tracked discontinuation in real time. Most relied on cross-sectional or retrospective designs, which are vulnerable to recall bias and often fail to capture switching behavior. This limitation is common in sub-Saharan African research, as highlighted in reviews from Kenya and Ghana, where poor data quality and lack of contraceptive calendars hinder accurate estimation of discontinuation pathways [89]. In contrast, DHS in countries like Egypt and Indonesia have more systematically used calendar data to monitor contraceptive dynamics over time [90, 91].

There were also important regional disparities within Nigeria. The review showed that most studies focused on the southwest zone of the country, leaving other zones barely captured. These findings emphasize the significance of context, as contraceptive use and discontinuation are deeply embedded in local norms, religious beliefs, and socio-political structures.

### Limitations

This review aimed to provide a comprehensive synthesis; however, limitations remain. Heterogeneity in how discontinuation was defined and measured, whether within 12 months, after method failure, or due to user dissatisfaction, complicates cross-study comparison. Furthermore, limited qualitative studies in some regions reduce the ability to interpret discontinuation within users’ lived experiences, a gap that has also been noted in FP research across sub-Saharan Africa [29]. Nevertheless, the findings offer critical insights into method-specific continuation and discontinuation patterns and underscore the need for comprehensive contraceptive counselling, improved follow-up systems, and user-centered approaches to FP in Nigeria and similar settings.

## Conclusion

The implications of these findings for reproductive health policy and programming are substantial. The high rates of discontinuation among users of short-acting methods emphasize the importance of improving client-provider interactions, especially at initiation. Government health authorities should establish and enforce national FP counselling standards. Donor organizations should finance provider training, counselling tools, follow-up systems, and commodity procurement; and non-governmental organizations should support community education, service delivery, and demand generation interventions that prioritize social norm change. State governments should ensure counter funding for contraceptive commodities, while facility managers provide supportive supervision to maintain counselling quality and service readiness. Healthcare providers are responsible for implementing these standards through structured, client-centered pre-use counselling that clearly explains side effects and sets realistic expectations. For condoms, product designers should prioritize comfort and sensation, incorporating user-centered innovations such as ultra-thin materials, varied sizes, and enhanced lubrication to reduce discontinuation and increase consistent use.

## Data Availability

All data generated or analyzed during this study are included in this published article.
